# A urinary assay for mutation and methylation biomarkers in the diagnosis and recurrence prediction of non-muscle invasive bladder cancer patients

**DOI:** 10.1186/s12916-023-03065-5

**Published:** 2023-09-19

**Authors:** Hai Huang, Ao Liu, Yiming Liang, Yaqun Xin, Jiacheng Liu, Yining Hao, Da Huang, Lu Chen, Wei Li, Guangliang Jiang, Yuhua Huang, Yaoting Xu, Jie Zhang, Tonghui Ma, Danfeng Xu, Yi Gao

**Affiliations:** 1 Department of Urology, Ruijin Hospital, Shanghai Jiaotong University School of Medicine, 197 Ruijin Road No.2, Shanghai, 200025 China; 2 Hangzhou Jichenjunchuang Medical Laboratory, Co., Ltd., Hangzhou, 310000 China; 3 Department of Urology, the First Affiliated Hospital of Soochow University, Suzhou, 215006 China; 4 Department of Urologic Surgery, Shanghai Fourth People`S Hospital Affiliated to Tongji University, No.1279, Sanmen Road, Shanghai, 200081 China; 5 Department of Translational Medicine, Genecn-Biotech. Co., Ltd., Hangzhou, 310000 China

**Keywords:** Non-muscle invasive bladder cancer, Cystoscopy, Mutation, Methylation, Diagnosis, Recurrence surveillance

## Abstract

**Background:**

Currently, the clinical strategy for diagnosis of non-muscle invasive bladder cancer (NMIBC) such as cystoscopy and cytology are invasive and/or with limited accuracy. OncoUrine, a urinary assay for mutation and methylation biomarkers, have showed a high accuracy in the detection of upper tract urinary carcinoma (UTUC) patients with hematuria. The aim of this study is to evaluate the performance of OncoUrine in diagnosis of NMIBC patients.

**Methods:**

In this multicenter prospective study, a total of 203 patients were enrolled, including 60 patients present with hematuria and 143 NMIBC patients under recurrence surveillance. Urine samples were collected before cystoscopy to undergo OncoUrine test. OncoUrine performance was calculated compared to clinical standard methods in hematuria cohort and recurrence surveillance cohort, respectively. Furthermore, NMIBC patients were followed up with a median time of 20.5 months (range 0.03 to 24.03 months) to assess the predictive value of OncoUrine during recurrence monitoring.

**Results:**

For bladder cancer diagnosis, OncoUrine tested 47 samples and achieved a sensitivity/specificity/positive predictive value (PPV)/negative predictive value (NPV) of 80% (95% CI 44.2–96.5)/91.9% (95% CI 77.0–97.9)/72.7% (95% CI 39.3–92.7)/94.4% (95% CI 80.0–99.0) (kappa value 69.4%, 95% CI 44.4–94.3), indicating 72.3% of unnecessary cystoscopy. For recurrence diagnosis, OncoUrine tested 93 samples, and the sensitivity/specificity/PPV/NPV was 100% (95% CI 59.8–100.0)/68.2% (95% CI 57.1–77.7)/22.9% (95% CI 11.0–40.6)/100% (95% CI 92.3–100.0) (kappa value 27.0%, 95% CI 11.1–42.8), indicating 62.4% of spared cystoscopy. What is more, OncoUrine correctly predicted 80% (20/25) of final recurrence with 12/25 (48%) patients who were OncoUrine positive, but cystoscopy negative was followed with recurrence during follow-up. The test result of OncoUrine was also found significantly correlated with recurrence free survival (RFS) of NMIBC patients (median 34.4-month vs unreached; HR 6.0, 95% CI 2.7–13.5, *P* < 0.0001).

**Conclusions:**

OncoUrine showed potential value to reduce the frequency of unnecessary cystoscopy and the healthcare cost of bladder cancer patients. Patients with positive test results represented a population who were at high risk of recurrence and thus should be subject to frequent surveillance to ensure timely detection of any potential recurrence. This study has been registered in ClinicalTrials.gov with the number NCT04994197 posted on August 2021.

**Supplementary Information:**

The online version contains supplementary material available at 10.1186/s12916-023-03065-5.

## Background

Bladder cancer is the 10th most common cancer and the most common malignancy of the urinary tract, with approximately 75% initially diagnosed as non-muscle invasive bladder cancer (NMIBC) [1, 2]. During follow-up, more than half of patients with NMIBC recur within 5 years and about 20% will progress to muscle invasive bladder cancer (MIBC) [3, 4]. Therefore, an accurate diagnosis and a strict surveillance is crucial for the decision-making of NMIBC management.

Currently, the gold standard method of diagnosis and surveillance of NMIBC is dependent on cystoscopy and confirmed with pathology. Cystoscopy has an overall high sensitivity but a limited specificity, especially in low-grade (LG) and carcinoma-in-situ (CIS) tumors [5–7]. Cytology, frequently used as adjunct to cystoscopy, is highly specific but its sensitivity is only 15–75% [8–11]. In clinical practice, patients with suspected symptoms typically undergo a comprehensive assessment that includes imaging, cytology, and cystoscopy for thorough evaluation to identify any suspicious areas of tumors. Pathological assessment will be performed for further confirmation if any suspicious tissues are detected during cystoscopy [12].

The high recurrence rate of NMIBC lead to extensive follow-up schemes with regular surveillance cystoscopies. Follow-up with invasive cystoscopies maintained for years would impose great negative impacts on patients’ quality of life. Therefore, the current challenge in bladder cancer management is to develop a reliable non-invasive method to improve the accuracy of selecting any suspicious patients and detecting any risk of recurrence. For this purpose, several urinary biomarkers have been developed to reduce the frequency of unnecessary cystoscopy. However, none of them has been incorporated in the current clinical guidelines because of insufficient sensitivity or negative predictive value (NPV) [13–18]. For instance, NMP22, BTA, and FISH are FDA-approved urinary tests for initial diagnosis and surveillance of bladder cancer despite an overall sensitivity of 57–82% and NPV of 21–48% [19]. One of the well-studied DNA methylation biomarkers EpiCheck presented a sensitivity of 68.2% in NMIBC follow-up and a sensitivity of 83% in upper tract urinary carcinoma (UTUC) diagnosis and 51% in bladder cancer diagnosis, respectively [13, 20].

OncoUrine is a urinary test composed a panel of 17 gene mutation and one gene methylation biomarkers. It has been shown to outperform either the mutation or the methylation biomarker alone and demonstrated a sensitivity of 92.2% and an NPV of 94.1% in our previous study on UTUC patients with hematuria [21]. In this study, a multicenter, prospective cohort of bladder cancer were enrolled to investigate the performance of OncoUrine in the diagnosis of NMIBC patients.

## Methods

### Study population

From June 2021 to March 2022, eligible patients were enrolled from 3 centers, with 142 patients (42 in the bladder cancer diagnosis cohort and 100 in the recurrence diagnosis cohort, respectively) from the main center Ruijin Hospital Shanghai Jiaotong University School of Medicine, 28 patients (8 and 20, respectively) from the First Affiliated Hospital of Soochow University, and 33 patients (10 and 23, respectively) from Shanghai Fourth People’s Hospital affiliated to Tongji University. The study was approved by the Institutional Ethical Committee and registered on ClinicalTrials.gov (NCT04994197). Informed consent was obtained from all patients.

Patients were included if they present with hematuria or previously diagnosed as NMIBC undergoing transurethral resection of bladder tumor (TURBT), are ≥ 18 years old, and are able to give written consent and provide a minimum of 100 ml urine before cystoscopy. Exclusion criteria were inadequate urine samples, failed OncoUrine test, and previously diagnosed as MIBC or other urinary tract cancers. Patients in the follow-up were included if a conclusive recurrence status was obtained within the follow-up time, and those missing during follow-up were excluded.

The primary endpoint was to determine the diagnostic characteristics of OncoUrine in bladder cancer detection and recurrence diagnosis of NMIBC. Diagnosis involves a combination of imaging cystoscopy and pathology. Recurrence was defined by cystoscopy and confirmed with pathological test. All procedures described in this study were in accordance with national and institutional ethical standards and the Declaration of Helsinki.

### Sample collection and DNA extraction

Prior to cystoscopy, 100 ml urine sample was collected from each patient. The tests were blinded to the clinical data of the patients. Urine samples were processed within 2 h and centrifuged at 2000 g for 10 min. The resulting pellet was washed once with phosphate-buffered saline and stored at – 80 °C until DNA extraction. DNA extraction was performed using the DNA reaction kit (QIAamp DNA Mini Kit 250, QIAGEN). DNA concentration was measured using the Qubit dsDNA HS Assay Kit (Thermo Fisher Scientific).

### Mutation sequencing analysis

The DNA sequencing library was prepared by PCR amplification with mixed DNA, primers, and polymerase. The multiplex PCR primers were provided in Supplementary Table 2. The library was purified and quantified before prepared for sequencing. DNA was then subjected to high-throughput sequencing on the Ion Proton system (Thermo Fisher Scientific) and sequencing analysis as described previously [21]. Briefly, sequencing data was obtained and underwent rigorous quality control to filter out low-quality reads and remove adapter sequences. After trimming and filtering, reads were aligned to the hg19 reference genome using Bowtie2 (version 2.2.4) to identify genetic variations. The identified variations were further annotated to determine the potential functional consequences. In this study, mutation frequency of 0.5% for all mutations and 3% for *TERT* C228T and *TP53* all sites were set as thresholds for variant detection. A cutoff of >  = 20 supporting unduplicated reads was used as cutoff to distinguish between the detected and undetected variants. The genes included in the panel was listed in Supplementary Table 2.

### Methylation analysis

Methylation-specific quantitative PCR was performed to evaluate the methylation status of the *ONECUT2* gene. DNA extracted from urine samples were subjected to bisulfite conversion following the manufacture’s protocol (EZ DNA Methylation-Lightning™ Kit, Zymo, USA). Subsequently, qPCR was carried out to amplify either methylated or unmethylated DNA sequences. The testing signals for methylation analysis were collected using the FAM channel, while the control signals were captured using the VIC channel. CT values were obtained to represent the relative quantity of methylated and unmethylated DNA. Methylation score was determined by calculating the delta CT (△Ct) values as △Ct = Ct FAM-Ct VIC. To classify the methylation status, a cutoff of △Ct = 8 was set. If the △Ct > 8 or if there was no signal detected in the FAM channel, the methylation test was considered negative.

### Statistical analyses

Statistical analysis was carried out using the GraphPad software (GraphPad Prism 8.2.1, San Diego, CA, USA) and R statistical software (version 3.6.1). Categorical data was compared by Fisher’s exact test, and continuous data was compared by Mann–Whitney test or unpaired *t*-test between groups. kappa test was used to evaluate the performance accuracy. Kaplan–Meier analysis was performed to compare the recurrence free survival rate between groups using a log-rank test. *P* values at *p* < 0.05 was considered statistically significant.

## Results

### Workflow of the study

**Fig. 1:**
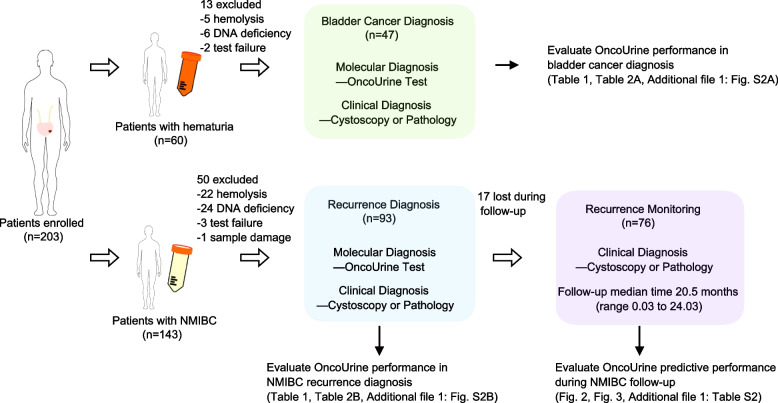
The workflow of the study

The detailed schematic description of the study was presented in Fig. 1 and Additional file 1: Fig. S1. A total of 203 patients including 60 patients with hematuria in the bladder cancer diagnosis cohort and 143 patients with NMIBC in the recurrence diagnosis cohort were enrolled. Among those, 13 patients (5 because of hemolysis, 6 because of DNA deficiency, and 2 because of test failure) were excluded from the 60 hematuria patients, and 50 patients (22 because of hemolysis, 24 because of DNA deficiency, 3 because of test failure and 1 because of sample damage) were excluded from 143 NMIBC patients. All patients underwent standard cystoscopies and OncoUrine test. An OncoUrine test result was defined positive if either mutation or methylation test was positive. The performance of OncoUrine was evaluated in the bladder cancer diagnosis and recurrence diagnosis cohort, respectively. Furthermore, patients with NMIBC were further followed up with a median time of 20.5 months (range 0.03 to 24.03 months), and the predictive value of OncoUrine during follow-up was also evaluated.

### OncoUrine performance for bladder cancer diagnosis

The patients’ characteristics of the bladder cancer diagnosis cohort were summarized in Table 1 and in Additional file 2: File S1. 61.7% (29 patients) of them were male with 66.0% were age ≥ 60 years old. Ten patients were diagnosed as malignant bladder cancer among which 6 were high grade (HG) tumors, and 37 were diagnosed as benign disease.

**Table 1:** Patients’ characteristics in this study

Clinical parameters	Bladder cancer diagnosis		Recurrence diagnosis	
	**Bladder cancer**	**Benign**	**Recurrence**	**Non-recurrence**
**Age (years)**				
< 60	1 (10%)	15 (40.5%)	2 (25%)	20 (23.5%)
≥ 60	9 (90%)	22 (59.5%)	6 (75%)	65 (76.5%)
**Gender**				
Male	8 (80%)	21 (56.8%)	6 (75%)	58 (68.2%)
Female	2 (20%)	16 (43.2%)	2 (25%)	27 (31.8%)
**Smoke status**				
Never	3 (30%)	17 (46%)	5 (62.5%)	43 (50.6%)
Current	1 (10%)	5 (14%)	2 (25%)	20 (23.5%)
Unknown	6 (60%)	15 (40%)	1 (12.5%)	22 (25.9%)
**Grade**				
Low	2 (20%)	NA	3 (37.5%)	NA
High	6 (60%)	NA	2 (25%)	NA
Unknown	2 (20%)	NA	3 (37.5%)	NA
**TNM**				
Ta	2 (20%)	NA	4 (50%)	NA
T1	5 (50%)	NA	0 (0%)	NA
T2	1 (10%)	NA	1 (12.5%)	NA
Unknown	2 (20%)	NA	3 (37.5%)	NA
**CIS**				
Yes	2 (20%)	NA	0 (0%)	NA
No	6 (60%)	NA	6 (75%)	NA
Unknown	2 (20%)	NA	2 (25%)	NA
**Tumor number**				
Single	4 (40%)	NA	3 (37.5%)	NA
Multiple	5 (50%)	NA	2 (25%)	NA
Unknown	1 (10%)	NA	3 (37.5%)	NA
**Tumor size**				
< 1.5 cm	2 (20%)	NA	0 (0%)	NA
≥ 1.5 cm	6 (60%)	NA	3 (37.5%)	NA
Unknown	2 (20%)	NA	5 (62.5%)	NA
**Time from last TURBT to urine collection (month; median range)**			10.3 (3.9–100.5)	9.4 (0.3–102.7)
**Number of confirmed recurrence**				
Single			3 (37.5%)	60 (70.6%)
Multiple			4 (50%)	22 (25.9%)
Unknown			1 (12.5%)	3 (3.5%)
**Treatment type**				
BCG			1 (12.5%)	15 (17.7%)
Bicin-kinds			6 (75%)	49 (57.7%)
GC			0 (0%)	12 (14.1%)
Others			1 (12.5%)	3 (3.5%)
Unknown			0 (0%)	6 (7%)

OncoUrine was positive in 8/10 tumors (Additional file 1: Fig. S2A; Additional file 2: File S2). No differences in age or gender were observed between OncoUrine positive and negative patients (Additional file 1: Table S1A). Compared to pathology, OncoUrine yielded a sensitivity of 80% (95% CI 44.2–96.5), a specificity of 91.9% (95% CI 77.0–97.9), PPV of 72.7% (95% CI 39.3–92.7), and NPV of 94.4% (95% CI 80.0–99.0), respectively (kappa value 69.4%, 95% CI 44.4–94.3). Given that OncoUrine identified 34 true negatives, it implied that OncoUrine could have a great potential to reduce the number of cystoscopies by 72.3% (34/47) (Table 2).

**Table 2:** The performance of OncoUrine for diagnosis in bladder cancer (bladder cancer diagnosis)

OncoUrine	Pathology		
	**Positive**	**Negative**	**Total**
**Positive**	8	3	11
**Negative**	2	34	36
**Total**	10	37	47
	n/N	% (95% CI)	
**Sensitivity**	8/10	80.0 (44.2–96.5)	
**Specificity**	34/37	91.9 (77.0–97.9)	
**PPV**	8/11	72.7 (39.3–92.7)	
**NPV**	34/36	94.4 (80.0–99.0)	
**Kappa value**		69.4 (44.4–94.3)	
**Spared Cystoscopy**	34/47	72.3	

### OncoUrine performance for recurrence diagnosis in NMIBC

Among the 93 patients included in the recurrence diagnosis analysis (Table 1; Additional file 2: File S1), 68.9% (64 patients) of them were male with 76% were age ≥ 60 years old. Eight patients were diagnosed as recurrence, with 3 were LG and 2 were HG tumors. OncoUrine was positive in 35 samples and negative in 58 samples (Additional file 1: Fig. S2B; Additional file 2: File S2). OncoUrine positive patients had a longer time since last TURBT (median 12.25-month vs 7.85-month; *P* = 0.032) and a higher number of previously confirmed recurrence (mean 2.18 vs 1.38; *P* = 0.018) than those with negative test results (Additional file 1: Table S1B).

The sensitivity, specificity, PPV, and NPV of OncoUrine was 100% (95% CI 59.8–100.0), 68.2% (95% CI 57.1–77.7), 22.9% (95% CI 11.0–40.6), and 100% (95% CI 92.3–100.0), respectively (kappa value 27.0%, 95% CI 11.1–42.8). Fifty-eight true negatives were identified by OncoUrine, which indicated that OncoUrine could potentially reduce 62.4% (58/93) of cystoscopies in patients under recurrence surveillance (Table 3).

**Table 3:** The performance of OncoUrine for diagnosis in bladder cancer (recurrence diagnosis)

OncoUrine	Pathology		
	**Positive**	**Negative**	**Total**
**Positive**	8	27	35
**Negative**	0	58	58
**Total**	8	85	93
	n/N	% (95% CI)	
**Sensitivity**	8/8	100.0 (59.8–100.0)	
**Specificity**	58/85	68.2 (57.1–77.7)	
**PPV**	8/35	22.9 (11.0–40.6)	
**NPV**	58/58	100.0 (92.3–100.0)	
**Kappa value**		27.0 (11.1–42.8)	
**Spared cystoscopy**	58/93	62.4	

### OncoUrine performance for recurrence prediction during NMIBC follow-up

**Fig. 2:**
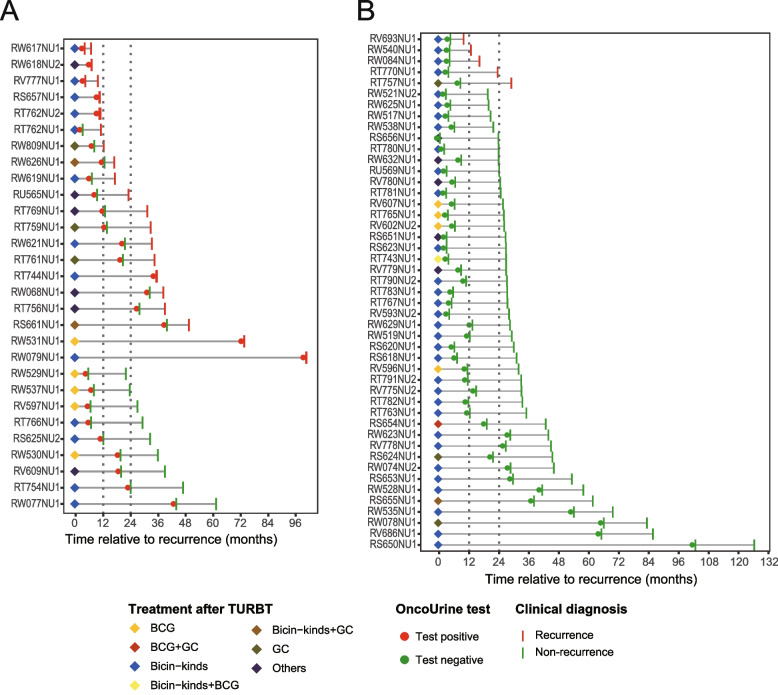
The longitudinal representation of patients with OncoUrine test results. Patients with positive and negative test results were represented in **A** and **B**, respectively. Red circle indicates a positive test result and green circle indicates a negative test result. Diamond filled with colors indicates different treatments after TURBT. Patients with recurrence and non-recurrence during follow-up were shown by red bar and green bar, respectively. The horizon lines indicate the month between last TURBT and recurrence

Next, we evaluated the recurrence predictive value of OncoUrine during follow-up. Among the 93 NMIBC patients, 17 patients were lost during follow-up and thus excluded for analysis. The recurrence status of the remaining 76 patients were recorded (Additional file 2: File S3). Patients were followed up with a median time of 20.5 months (range 0.03 to 24.03 months). Twenty-five patients were found recurred during follow-up confirmed with cystoscopy and pathological test (median follow-up time: 8.0-month, range 0.03 to 20.4 months), while 51 were found with non-recurrence (median follow-up time: 20.5-month, range 15.07 to 24.03 months) (*P* < 0.0001). The longitudinal representation of OncoUrine positive and negative patients during follow-up was shown in Fig. 2.

**Fig. 3:**
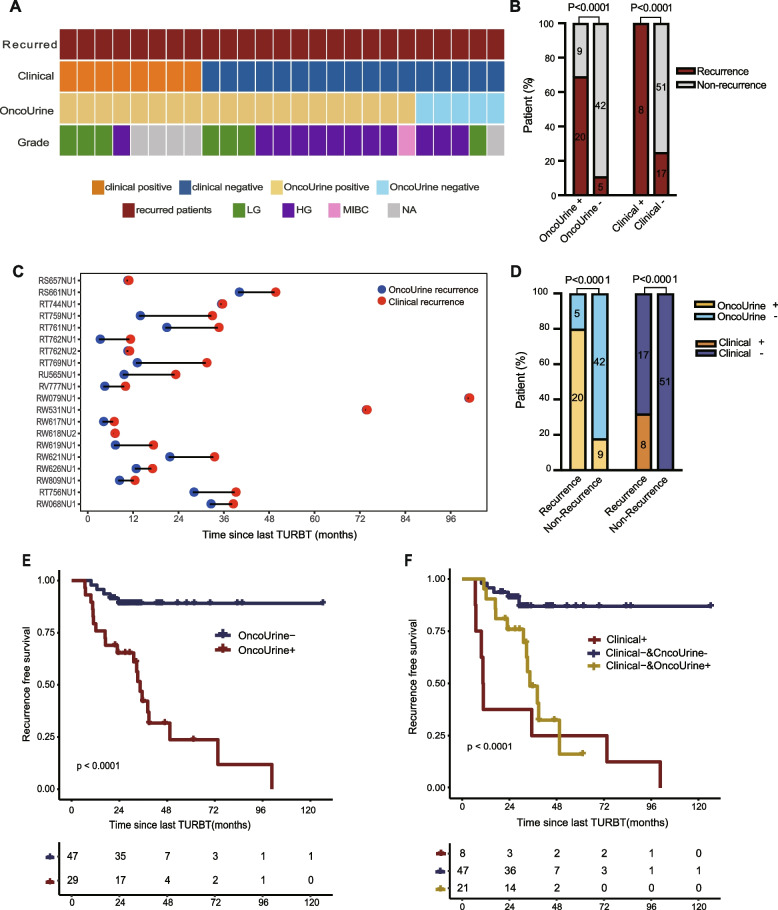
The performance of OncoUrine in recurrence prediction during follow-up. **A** The clinical and OncoUrine results of the recurred patients during follow-up. The correlation of recurrence during follow-up with test and clinical results was shown in **B** and **D**, respectively. **C** The lead time of recurrence between OncoUrine test results and clinical confirmed recurrence. The term “OncoUrine recurrence” refers to a positive test result at recruitment while “Clinical recurrence” refers to the tumor recurrence subsequently confirmed by cystoscopy and pathology. The *x* axis indicates the time since TURBT. The recurrence free survival plot showed that a negative OncoUrine test was associated with a better outcome (**E**) and adding OncoUrine result to clinical diagnosis could further stratify patients’ risk (**F**). Statistical significance was calculated by log-rank tests. LG, low grade. HG, high grade. MIBC, muscle invasive bladder cancer. OncoUrine + , OncoUrine positive. OncoUrine -, OncoUrine negative. Clinical + , clinical positive. Clinical -, clinical negative

Surprisingly, OncoUrine result at the time of recurrence diagnosis correctly predicted 20 of the 25 (80%) patients with recurrence during follow-up, while clinical standards detected 8 of them (32%). The 12 samples with positive test results but negative cystoscopies were followed with recurrence after a time ranging from 4.07 to 19.07 months. To be noted, 3 of the 12 samples were diagnosed as LG, 8 were HG, and 1 was MIBC at the time of recurrence during follow-up, indicating the ability of OncoUrine to predict recurrence even in LG tumors (Fig. 3A). We found a significant correlation between OncoUrine test results and recurrence during follow-up. The recurrence rate was 69.0% (20/29) in OncoUrine positives vs 10.6% (5/47) in OncoUrine negatives (*P* < 0.0001). Meanwhile, OncoUrine positives accounted for 80% (20/25) in patients with recurrence but was only 17.7% (9/51) in those with non-recurrence (*P* < 0.0001) (Fig. 3B; D).

Compared to clinical confirmed recurrence, OncoUrine could predict recurrence with a median lead time of 5.68 months (range 0.03 to 19.06 months; *P* = 0.11; Fig. 3C). However, the time difference was not statistically significant which might be due to the limited sample size (more detailed predictive performance of OncoUrine was shown in Additional file 1: Table S2).

Furthermore, we explored the prognostic value of OncoUrine in NMIBC patients. Recurrence free survival (RFS) was measured from the date of last TURBT to verified first pathological recurrence during follow-up. We found that OncoUrine positive patients had a significantly shorter RFS than negative ones (median 34.4-month vs unreached; HR 6.0, 95% CI 2.7–13.5, *P* < 0.0001; Fig. 3E). This was the same when compared clinical positive and negative patients (median 10.7-month vs unreached; HR 10.8, 95% CI 2.9–40.6, *P* = 0.0004; data not shown). However, we found that implementing OncoUrine results could further stratify patients into different risk subgroups. For patients with clinical negative diagnosis, those with OncoUrine positive results had worse RFS than those with OncoUrine negative results (median 34.4-month vs unreached; *P* < 0.0001; Fig. 3F).

Collectively, our results here showed that OncoUrine exhibited promising improvements of recurrence prediction compared with clinical standards, indicating its potential application in clinical practice during NMIBC monitoring.

### Potential implementation of OncoUrine in clinic practice for NMIBC management

**Fig. 4:**
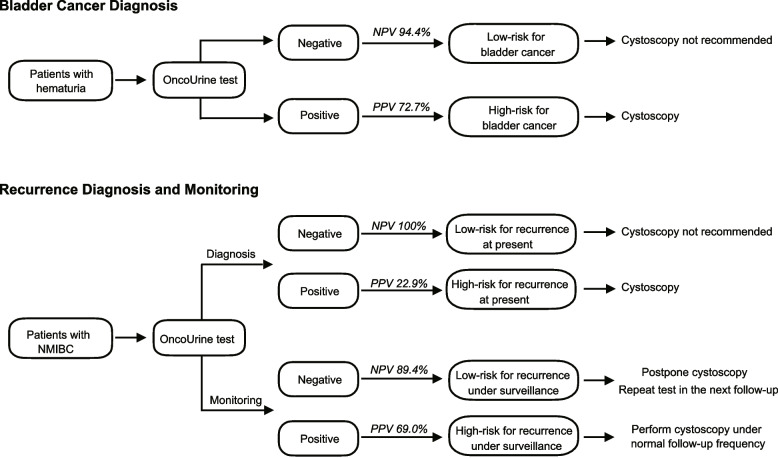
A proposed model to incorporate OncoUrine in clinical practice. **A** The potential application of OncoUrine in bladder cancer diagnosis. **B** The potential application of OncoUrine in recurrence diagnosis and monitoring. In both clinical settings, patients with negative test results have low risk of developing bladder cancer or recurrence and cystoscopy could be avoided. While patients with positive test results should undergo confirmatory cystoscopy

Based on our results above, we proposed a model to possibly incorporate OncoUrine into clinic practice for diagnosis and recurrence monitoring in NMIBC (Fig. 4).

In the setting of bladder cancer diagnosis, patients present with hematuria would receive OncoUrine test. For patients with positive test result, confirmatory cystoscopy should be performed following clinical guidelines. However, based on the high NPV, those with negative test results are thought to have a low likelihood diagnosed as malignant bladder cancer and thus cystoscopies are not recommended.

In the setting of recurrence surveillance, NMIBC patients under regular surveillance are included to undergo OncoUrine test. For patients with positive test results, confirmed cystoscopy under normal follow-up frequency should be performed. While a negative test result could confidently indicate a non-recurrence at present and a low risk to recurrence in the near future. For these low-risk recurrence patients, cystoscopies can be postponed, and surveillance frequency can be reduced.

## Discussion

While there are significant differences in the mutational spectra between UTUC and bladder cancer, we developed the OncoUrine urinary test based on a multi-marker approach. The test panel included hotspot mutations in 17 genes as well as the methylation biomarker ONECUT2 to best cover the shared genetic alterations in UTUC and bladder cancer (primers were listed in Additional file 1: Table S3). The performance of OncoUrine has been validated in UTUC and showed good performance in discriminate malignant from benign disease in UTUC patients with hematuria in our previous publication [21]. In this study, we further validated the performance of OncoUrine in the diagnosis and surveillance in bladder cancer to expand its clinical application. OncoUrine demonstrated a sensitivity of 80% (95% CI 44.2–96.5) and NPV of 94.4% (95% CI 80.0–99.0) in bladder cancer diagnosis and a sensitivity of 100% (95% CI 59.8–100.0) and NPV of 100% (95% CI 92.3–100.0) in recurrence diagnosis, respectively. This added evidence of the potential application of OncoUrine in bladder cancer clinical management.

Currently, a great challenge in bladder cancer management is the frequent and long-term surveillance with invasive cystoscopy. In patients with a history of NMIBC, the prevalence is high and a biomarker with a high sensitivity and NPV is mandatory to ensure a low chance of missing recurrent tumors and to help rule out patients who could avoid unnecessary cystoscopy. Up to now, although several urinary biomarkers aiming to reduce cystoscopy under NMIBC surveillance were investigated, none of them have been applied in clinic due to insufficient sensitivity or NPV. For example, several studies have reported that ONECUT2 methylation combined with other biomarkers can effectively discriminate bladder cancer from benign disease [22–24]. A two-methylation biomarker assessing ONECUT2 and VIM called UriFind achieved a sensitivity of 89% in recurrent tumors [14]. Consistently, ONECUT2 methylation alone in our study was present in 8 of 10 bladder cancer patients and 10 of 10 recurrent patients (Additional file 1: Fig. S2), indicating the potential role of ONECUT2 methylation in bladder cancer diagnosis. The test performance of distinct gene mutations has also been evaluated in bladder cancer. Uromonitor-V2 based on the assay of three gene mutation*s* (*TERT*, *FGFR3*, and *KRAS*) showed a sensitivity of 93% and an NPV of 95% in detecting NMIBC recurrence [25]. In our mutation analysis, we selected a set of 17 genes including the three used in Uromonitor-V2 and showed that *TERT*, *TP53*, and *PIK3CA* were most frequently mutated genes, which was consistent as previously reported [26–28]. However, using the 17 gene set only yielded a sensitivity of 50% (Additional file 1: Fig. S2B). This discrepancy in sensitivity may be attributed to genomic differences due to races since the population in Uromonitor-V2 was sourced from European patients. Compared to the above studies, OncoUrine integrated both methylation and mutation biomarkers yielded 80% (95% CI 44.2–96.5) sensitivity and 94.4% (95% CI 80.0–99.0) NPV in bladder cancer diagnosis and 100% (95% CI 59.8–100.0) sensitivity and 100% (95% CI 92.3–100.0) NPV in recurrence diagnosis, demonstrating great potential to reduce 72.3% and 62.4% of cystoscopies, respectively. In the future, a smaller panel with fewer genes combined with clinical features can be investigated to further improve the test performance and reduce patient costs. Also, more efforts to help urine collection and shipment at home can be made to reduce patients’ visits to hospital.

The OncoUrine test yielded a low PPV in both diagnosis and follow-up in the recurrence cohort, which was 22.9% and 69%, respectively. The low PPV value would indicate a high rate of false positives with the prevalence of 8.6% (8/93) in the recurrence diagnosis cohort. Consequently, there was risk of patient misdiagnosis and with unreliable recurrence predictions, which would cause patient anxiety as well as extra healthcare costs. Therefore, the low PPV calls for caution in interpreting positive test results and require further confirmatory tests to verify the presence of recurrence. Further improvements such as validating in a larger cohort or implementing additional biomarkers to improve the overall test performance are required in the future. Under surveillance, a false positive test result was thought to have predictive value for future tumor recurrence [29–31]. Indeed, 48% (12/25) of the recurrence during follow-up were detected by OncoUrine but were missed by clinical standard-of-care in our study. The recurrence rate in patients with false-positive test results was significantly higher than that in patients with true-negative results (57.1% vs 10.6%; *P* = 0.0001; Additional file 1: Table S4). This implies that patients with positive test results under surveillance could represent a population who were at high-risk of recurrence and confirmatory cystoscopy is required during follow-up. However, patients with negative test results could postpone cystoscopy and repeat test in the next follow-up since the risk to recurrence is low.

Limitations of this study included a limited sample size that the performance of OncoUrine could not be further analyzed between LG and HG tumors. We also did not add multiple time points during follow-up and patient with positive OncoUrine tests could be followed-up with longer time to verify their recurrence status. Future prospective study with a larger sample size and longer follow-up period with multiple test time points should be performed for further validation.

## Conclusions

Taken together, this study demonstrates the promising applications of OncoUrine in clinic management of NMIBC. The employment of OncoUrine will help reduce the frequency of cystoscopy and thus patients’ healthcare expenses. Patients with positive test results represented a population who were at high risk of recurrence and thus should be subject to frequent surveillance to ensure timely detection of any potential recurrence.

### Supplementary Information

**Additional file 1: Fig. S1.** A detailed schematic of the study. **Fig. S2.** The mutation landscape in the two cohorts. A. The mutation landscape and clinical characteristics in patients with hematuria. B. The mutation landscape and clinical characteristics in patients with NMIBC. **Table S1.** Patients’ characteristics between OncoUrine positive and negative groups. **Table S2.** The predictive performance of OncoUrine during follow-up. **Table S3.** The gene list and primers of the OncoUrine panel. Table S4. The recurrence rate during follow-up in patients with false positive OncoUrine test results and with true negative results.

**Additional file 2: File S1.** The clinical characteristics and OncoUrine test results of the patients in the bladder cancer diagnosis cohort, the recurrence diagnosis cohort, and the follow-up cohort, respectively. **File S2.** The mutational frequency and methylation levels of the OncoUrine test positive patients. **File S3.** The follow-up data with confirmed recurrence status of patients.

## Data Availability

The dataset supporting the conclusions of this article is included within the article and its additional files. The dataset supporting the conclusions of this article is available in the Genome Variation Map in National Genomics Data Center, Beijing Institute of Genomics, Chinese Academy of Sciences, and China National Center (https://bigd.big.ac.cn/gvm/getProjectDetail?Project=GVM000506) for Bioinformation, under accession number GVM000506.

## Abbreviations

CIS: Carcinoma in situ
HG: High grade
LG: Low grade
MIBC: Muscle invasive bladder cancer
NMIBC: Non-muscle invasive bladder cancer
NPV: Negative predictive value
PPV: Positive predictive value
RFS: Recurrence free survival
TURBT: Transurethral resection of bladder tumor
UTUC: Upper tract urinary carcinoma
