# Supplementary material for: A urinary assay for mutation and methylation biomarkers in the diagnosis and recurrence prediction of non-muscle invasive bladder cancer patients
Source: BMC Med. 2023 Sep 19;21:357. doi: 10.1186/s12916-023-03065-5 (PMC10510256; doi:10.1186/s12916-023-03065-5)
Supplement: Supplementary file 1 — Additional file 1: Fig. S1. A detailed schematic of the study. Fig. S2. The mutation landscape in the two cohorts. A. The mutation landscape and clinical characteristics in patients with hematuria. B. The mutation landscape and clinical characteristics in patients with NMIBC. Table S1. Patients’ characteristics between OncoUrine positive and negative groups. Table S2. The predictive performance of OncoUrine during follow-up. Table S3. The gene list and primers of the OncoUrine panel. Table S4. The recurrence rate during follow-up in patients with false positive OncoUrine test results and with true negative results. [file 12916_2023_3065_MOESM1_ESM.zip › Table S1R2.docx]

**Table S1. Patients’ characteristics between OncoUrine positive and negative groups**

**A: Bladder Cancer Diagnosis**

| **Characteristics** | **OncoUrine** | | | |  |
| --- | --- | --- | --- | --- | --- |
|  | **Positive** | | **Negative** | ***P* value** | |
| **Age (yrs; median)** | 68 | | 62 | 0.148 | |
| **Gender** |  | |  | 0.164 | |
| Male | 9 | | 20 |  | |
| Female | 2 | | 16 |  | |
| **Grade** |  |  | |  | |
| Low | 1 | 1 | |  | |
| High | 6 | 0 | |  | |
| NA | 4 | 35 | |  | |

**B: Recurrence Diagnosis**

| **Characteristics** | **OncoUrine** | | | |  |
| --- | --- | --- | --- | --- | --- |
|  | **Positive** | | **Negative** | ***P* value** | |
| **Age (yrs; median)** | 67 | | 67 | 0.245 | |
| **Gender** |  | |  | 0.172 | |
| Male | 21 | | 43 |  | |
| Female | 14 | | 15 |  | |
| **Grade** |  |  | |  | |
| Low | 3 | 1 | |  | |
| High | 2 | 0 | |  | |
| NA | 30 | 57 | |  | |
| **Time Since Last TURBT (month; median)** | 12.25 | 7.85 | | 0.032 | |
| **Number of Confirmed Recurrence (mean)** | 2.18 | 1.38 | | 0.018 | |
